# Simple IHC reveals complex MMR alternations than PCR assays: Validation by LCM and next‐generation sequencing

**DOI:** 10.1002/cam4.4832

**Published:** 2022-05-21

**Authors:** Kenji Amemiya, Yosuke Hirotsu, Yuki Nagakubo, Shunsuke Watanabe, Saki Amemiya, Hitoshi Mochizuki, Toshio Oyama, Tetsuo Kondo, Masao Omata

**Affiliations:** ^1^ Division of Genetics and Clinical Laboratory Yamanashi Cental Hospital Yamanashi Japan; ^2^ Genome Analysis Center Yamanashi Cental Hospital Yamanashi Japan; ^3^ Department of Pathology, School of Medicine University of Yamanashi Yamanashi Japan; ^4^ Department of Pathology Yamanashi Central Hospital Yamanashi Japan; ^5^ Department of Gastroenterology Yamanashi Central Hospital Yamanashi Japan; ^6^ Department of Gastroenterology The University of Tokyo Tokyo Japan

**Keywords:** heterogeneity, immunohistochemistry, MMR, MSI, next‐generation sequencing

## Abstract

Evaluation of the status of mismatch repair (MMR) in tumors is crucial for determining the application of immune checkpoint inhibitors (ICIs). Conventional PCR (MSI‐PCR) is the gold standard for confirming the MMR status. However, it requires visual confirmation and presents difficulties in determining MMR status. Immunohistochemistry (IHC) is a simple method and can confirming MMR protein expression in the whole tumor. We aim to investigate IHC is more suitable for evaluating MMR status in the tumor. We compared MSI‐PCR and IHC by testing 319 samples from 284 patients across 14 cancer types. In discordant cases, we performed laser‐capture microdissection and microsatellite instability assay by next‐generation sequencing (MSI‐NGS). The concordance rate between IHC and MSI‐PCR testing was 98.1% (313/319). Two reasons for these discrepancies were ambiguous MSI‐PCR results and heterogeneous MSI status within the tumor. Among six cases (1.9%), three were judged as MSI‐H by MSI‐PCR but with proficient MMR by IHC. The results of MSI‐NGS revealed microsatellite stable in these three cases. The remaining three cases, two of three were MSI‐H and one was MSS in whole tumor in MSI‐PCR. IHC showed a “mosaic” pattern containing both proficient MMR and deficient MMR portions by IHC in all three cases. We performed microdissection and MSI‐PCR and found intratumoral heterogeneity of MMR status. These results indicated the advantages of IHC and performed expanded samples (n = 1082) and two additional mosaic cases were identified. Our results clearly indicated that simple IHC is the best choice for determining MMR alterations in critical cases for ICIs treatment.

## Graphical abstract

We compared the results between MSI‐PCR and IHC and examined the reasons for discrepancies in results between these tests using laser capture microdissection (LCM) and MSI‐NGS. Our results showed that simple IHC is one of the best choice for determining precise MMR status and revealing mosaic pattern within tumor.

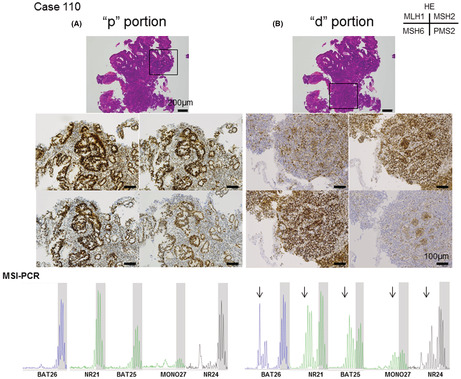

## 1 INTRODUCTION

Immuno‐oncology therapy has greatly affected the treatment options for various cancer types.^1^, ^2^, ^3^, ^4^, ^5^, ^6^, ^7^ In particular, the treatment of malignant melanoma with immune checkpoint inhibitors (ICIs) as first‐line therapy was a step forward.^8^ In May 2017, the results of the KEYNOTE‐164 and KEYNOTE‐158 trials led to the approval of pembrolizumab by the Food and Drug Administration for the treatment of unresectable or metastatic solid tumors expressing a biomarker referred to as microsatellite instability‐high (MSI‐H) or deficient mismatch repair (dMMR).^9^, ^10^

The status of mismatch repair (MMR) and microsatellite instability (MSI) can be determined using three different methods. Microsatellite instability‐polymerase chain reaction (MSI‐PCR) uses DNA extracted from tumor tissue to analyze five microsatellite loci. MSI at more than two loci out of five is defined as MSI‐H, that at one of five loci is defined as MSI‐low, and no instability at any of five loci is considered as microsatellite stable (MSS). MSI‐PCR testing can only be performed in well‐equipped institutions and requires some molecular expertise. Another method is MMR‐Immunohistochemistry (MMR‐IHC) using formalin‐fixed paraffin‐embedded (FFPE) tissue sections. IHC is widely used in general pathological laboratories. The complete absence of nuclear staining of least one of the four MMR proteins (MLH1, MSH2, MSH6, and PMS2) is defined as dMMR, and as proficient MMR (pMMR) when they are retained. MLH1 and PMS2 form a functional complex, and loss of MLH1 expression simultaneously leads to loss of PMS2 expression, whereas expression of MLH1 is not affected by PMS2.^11^, ^12^ MSH2 and MSH6 are similar; loss of MSH2 expression simultaneously leads to loss of MSH6 expression, but MSH2 expression is not affected by MSH6.^13^ MSI‐PCR and MMR‐IHC testing have been used for screening testing for Lynch syndrome.^14^, ^15^, ^16^, ^17^ In addition, we previously reported non‐hereditary right‐sided colon cases with MSI‐H as determined by MSI‐PCR.^18^, ^19^ Since then, there have been many reports on the association between IHC and PCR assays. The concordance rate between MSI‐PCR and MMR IHC testing is reported to be over 90%.^20^, ^21^, ^22^, ^23^ There are a few studies on the discordant results between the two methods^17^, ^24^, however it remains unclear of this discordance.

In this study, we performed a paired analysis of MSI‐PCR and MMR‐IHC across multiple‐organ samples (319 samples from 284 patients with 14 tumor types: Validation cohort) and tested the concordance between the two assays. Because we have developed a third method using next‐generation sequencing (NGS) named microsatellite instability assay by NGS (MSI‐NGS),^25^ we explored the reasons behind the discordant results of MSI‐PCR and MMR‐IHC in combination with laser capture microdissection (LCM).

## 2 MATERIALS AND METHODS

### 2.1 Patient and cohort groups (validation and expanded cohorts)

**FIGURE 1:**
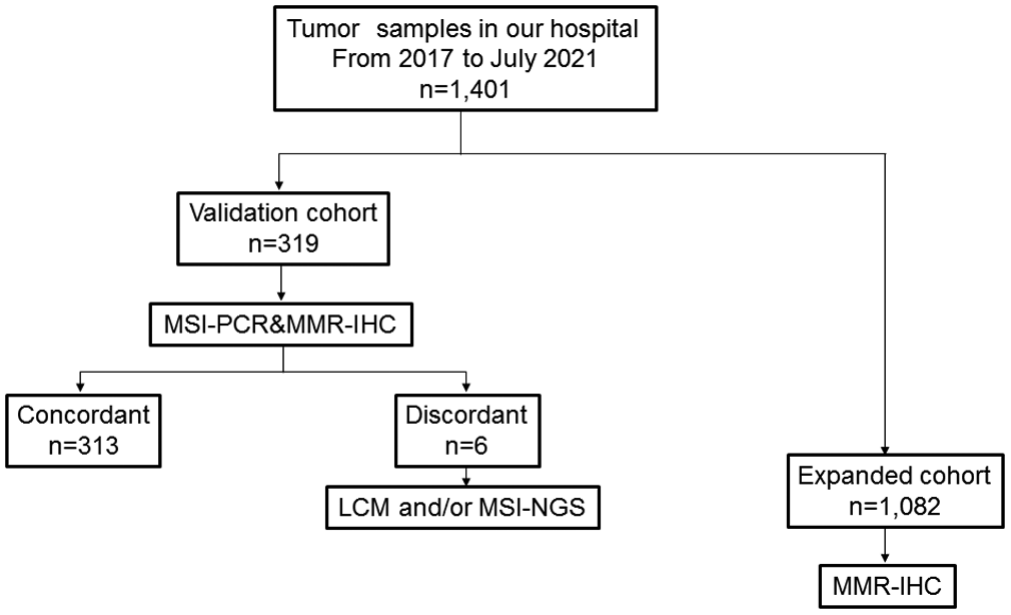
Flowchart of the Validation and Expanded cohorts in this study. Discordant cases of MSI‐H/pMMR were analyzed by MSI‐NGS. Partially discordant cases (mosaic pattern), namely pMMR and dMMR, were mixed (p/d MMR) and LCM and MSI‐NGS were performed

From September 2017 to May 2021, written informed consent was obtained from 1224 patients, and 1401 FFPE samples were available for this study (Figure 1). This study was approved by the institutional review board at our hospital (G2018–1, G2019–5). Written informed consent was obtained from all patients who participated in this study.

#### 2.1.1 Validation cohort

In 319 FFPE samples from 285 patients, both MSI‐PCR and MMR‐IHC were performed to assess the concordance and discordance between the two assays as the “Validation cohort.” We have developed a third method that uses MSI‐NGS.^25^ Using this approach, we explored the reasons behind the discordant results between MSI‐PCR and MMR‐IHC.

#### 2.1.2 Expanded cohort

Because the preliminary results indicated the advantages of MMR‐IHC, we further performed MMR‐IHC in 1082 samples from 939 patients as the “Expanded cohort.” (Figure 1).

### 2.2 DNA extraction

Tumor DNA was extracted from two 10‐μm‐thick sections of resected tissue and five 10‐μm‐thick sections of biopsy samples using an Agencourt Forma‐Pure DNA kit (Beckman Coulter) according to the manufacturer's protocol.^26^ DNA concentration was measured with a NanoDrop 2000 spectrophotometer (Thermo Fisher Scientific).

### 2.3 MSI‐PCR

MSI‐PCR was performed using an MSI diagnostic kit (FALCO Biosystems, Kyoto, Japan) and an MSI Analysis System v1.2 (Promega).^27^, ^28^

MSI‐PCR analysis was performed using five microsatellite loci (BAT‐25, BAT‐26, NR‐21, NR‐24, and MONO‐27) (FALCO and Promega kit) or BAT‐25, BAT‐26, D17S250, D2S123, and D5S346.^27^, ^28^ Microsatellite instability more than at two loci was defined as MSI‐H, while that at one locus was defined as MSI‐L, and no instability at any of the loci was defined as MSS.

### 2.4 MMR‐IHC

Four 3‐μm‐thick sections of FFPE samples were made for IHC analysis and stored at 37°C overnight. We used two auto‐staining systems. Protein expression was evaluated with mutL homolog 1 (MLH1) (M1; Ventana Medical Systems; Roche Group), mutS homolog 2 (MSH2) (G219‐1129; Ventana Medical Systems), mutS homolog 6 (MSH6) (44; Ventana Medical Systems), and postmeiotic segregation 1 homolog 2 (PMS2) (EPR3947; Ventana Medical Systems) antibodies using the Ventana BenchMark XT system (Roche). Protein expression was evaluated with MLH1 (ES05; Agilent), MSH2 (FE11; Agilent), MSH6 (EP49; Agilent), and PMS2 (EP51; Agilent) antibodies using the Autostainer Link48 (Agilent).^27^, ^28^ All sections were evaluated by a medical technologist (K.A.) and a pathologist (T.O). Tumors with completely absent nuclear staining of at least one of MLH1, MSH2, MSH6, or PMS2 were classified as dMMR.

### 2.5 LCM

Serial 10‐μm‐thick FFPE sections were made and stored at room temperature overnight in 1.5 ml sterilized tubes until DNA extraction. Three‐micrometer‐thick sections were then prepared and stained with hematoxylin–eosin (HE) to check sample adequacy and tumor cellularity. All slides were reviewed by T.O and K.A. In this study, tumor tissues showed a “mosaic” pattern of IHC in which pMMR and dMMR were mixed in a tumor (p/d MMR). These cases were subdivided by LCM using an Arcturus XT system (Thermo Fisher Scientific) and nucleic acid was extracted for re‐analysis.

### 2.6 MSI‐NGS

We previously reported an MSI‐NGS method that generates an MSI score (MSIcall).^25^ In brief, multiplex PCR was performed using an Ion AmpliSeq Microsatellite Instability Research Panel and Ion AmpliSeq Library Kit Plus (Thermo Fisher Scientific). This panel contains a single primer pool to amplify 76 microsatellite loci that are known to be affected by MSI.^29^, ^30^, ^31^ Primers were partially digested with FuPa reagent and then barcoded using Ion Xpress Barcode Adapters. After purification with Agencourt AMPure XP reagents (Beckman Coulter), the library concentration was determined using an Ion Library Quantitation Kit. Sequencing was performed using an Ion PI chip and Ion PI Hi‐Q Sequencing Kit on an Ion Proton Sequencer (Thermo Fisher Scientific). MSI score was calculated according to the homopolymer signal of each mapped forward and reverse read, and the mean of the homopolymer signal with normalization was used to examine the distance to that of the equivalent marker in the control. MSI score was calculated as a weighted normalized sum of the marker scores in the forward and reverse directions. The highest level of diagnostic accuracy of the MSI score of MSI‐NGS had a cutoff of 40.^25^ On the basis of the calculated MSI score, samples with an MSI score of more than 40 were judged as MSI‐H and samples with MSI scores of less than 40 were defined as MSS.

### 2.7 Bisulfite conversion and MLH1 methylation analysis

Bisulfite conversion was performed using MethylCode™ Bisulfite Conversion Kit (Thermo Fisher Scientific) according to the manufacturer's protocol. CT conversion reagent was added to 20 μl DNA samples and mixed by flicking the tube. Bisulfite process was conducted under following conditions: 10 min at 98°C and following with 150 min at 64°C and ended with a holding period at 4°C. After that, the binding buffer was mixed with bisulfite‐converted solution, and added to spin column. The spin column was washed with wash buffer and desulphonated by adding desulphonation buffer at 20 min. After washing the column, DNA was eluted by 10 μl elution buffer.

MLH1 methylation analysis was performed using EpiScope® MSP kit (Takara) and Epitect® PCR Control DNA Set (Qiagen) on a ViiA 7 Real‐Time PCR System (Thermo Fisher Scientific) according to the manufacturer's protocol. We compared sample DNA with bisulfite converted methylated and unmethylated human control DNA at amplification plot and melting curve plot. The PCR protocol was below: 95°C for 30 s, followed

by 45 cycles of 98°C for 5 s, 56°C for 30 s, and 72°C 60 s, and the following step was 95°C for 15 s, 60°C for 60 s, and 95°C 15 s.

### 2.8 Analysis of somatic variants of MMR genes using NGS

Library preparation was using an Ion AmpliSeq Library Kit Plus (Thermo Fisher Scientific), as previously described.^25^ Sequencing was performed using an Ion PI chip and Ion PI Hi‐Q Sequencing Kit on an Ion Proton Sequencer (Thermo Fisher Scientific). We analyzed somatic variants of MMR genes (MLH1, MSH2, MSH6, and PMS2) using in‐house colon cancer panel targeting 60 genes (2996 primer pairs). The sequence data were processed using standard Ion Torrent Suite™ Software (Thermo Fisher Scientific) on a Torrent Server. Raw signal data were analyzed using Torrent Suite version 5.10. After data analysis, variant calling was performed with filtering using the Ion Reporter Server System (Thermo Fisher Scientific), and buffy coat DNA was used as a control to detect variants in tumors.

## 3 RESULTS

### 3.1 Comparison of MSI‐PCR and MMR‐IHC in the validation cohort

MMR‐PCR and MSI‐IHC assays were performed in 319 FFPE samples in the Validation cohort. MSI‐PCR yielded 37 MSI‐H and 282 MSS cases (Tables 1, 2 and Table S1).

**TABLE 1:** Comparison of MSI‐PCR and MMR‐IHC in 319 cases *(Validation* cohort)

		MMR‐IHC			Total
		pMMR	p/dMMR	dMMR	
**MSI‐PCR**	**MSS**	**281**	**1**	**0**	**282**
	**MSI‐H**	**3**	**2**	**32**	**37**
	**Total**	**284**	**3**	**32**	**319**

Abbreviations: MMR‐IHC, mismatch repair‐immunohistochemistry; pMMR, proficient MMR; p/d MMR, proficient and deficient MMR; MSI‐PCR, microsatellite instability‐ polymerase chain reaction; MSS, microsatellite‐stable; MSI‐H, microsatellite instability high.

**TABLE 2:** Results of MSI‐PCR and MMR‐IHC of each cancer type in 319 cases (*Validation* cohort)

Cancer type	*n*	MSI‐PCR		MMR‐IHC		
		MSS	MSI‐H	pMMR	dMMR	p/dMMR
Gastric	89	72 (80.9%)	17 (19.1%)	72 (80.7%)	15 (17.0%)	2 (2.2%)
Colorectal	69	59 (85.5%)	10 (14.5%)	59 (85.5%)	10 (14.5%)	0
Breast	38	36 (94.7%)	2 (5.3%)	38 (100%)	0	0
Lung	21	19 (90.5%)	2 (9.5%)	20 (95.2%)	1 (4.8%)	0
Esophagus	19	19 (100%)	0	19 (100%)	0	0
Endometrium	17	13 (76.5%)	4 (23.5%)	12 (70.6%)	4 (23.5%)	1 (5.9%)
Ovary	12	12 (100%)	0	12 (100%)	0	0
Pancreas	10	10 (100%)	0	10 (100%)	0	0
Cervix	6	6 (100%)	0	6 (100%)	0	0
Bladder	6	4 (66.7%)	2 (33.3%)	4 (66.7%)	2 (33.3%)	0
Bile duct	3	3 (100%)	0	3 (100%)	0	0
Liver	3	3 (100%)	0	3 (100%)	0	0
Prostate	3	3 (100%)	0	3 (100%)	0	0
Small intestine	1	1 (100%)	0	1 (100%)	0	0
Other	22	22 (100%)	0	22 (100%)	0	0

Abbreviations: MMR, mismatch repair; pMMR, proficient MMR; dMMR, deficient MMR; p/d MMR, proficient and deficient MMR.

In 76 samples from eight organs and miscellaneous regions, no MMR abnormality was identified either by PCR or by IHC (Table 2). In the remaining six organs (gastric, colorectal, breast, lung, endometrium, and bladder: 240 samples), MMR abnormality was detected by either PCR or MMR‐IHC. IHC yielded three types: pMMR (*n* = 284), dMMR (*n* = 32), and mosaic p/dMMR (*n* = 3) (Table 2).

**FIGURE 2:**
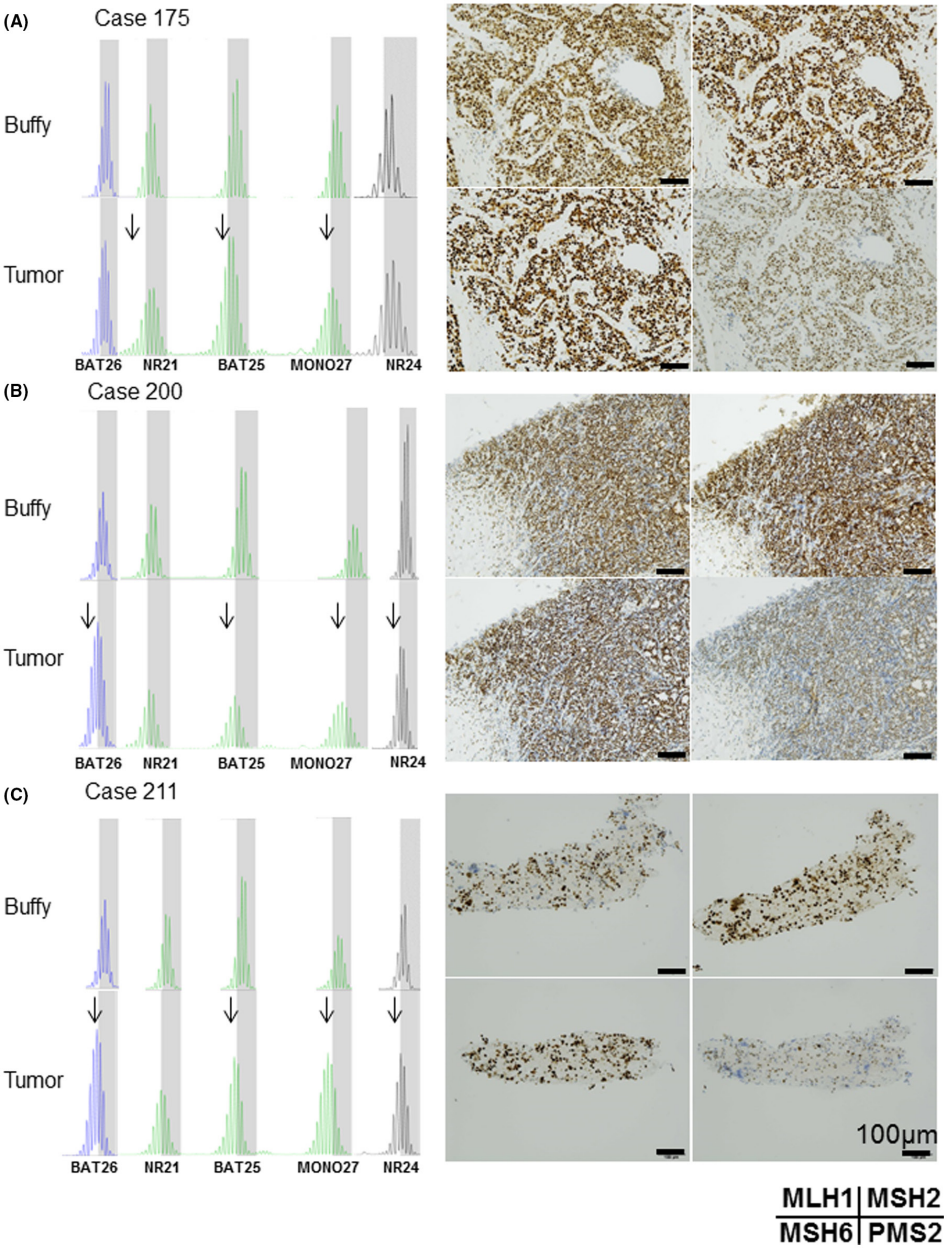
MSI‐PCR and MMR‐IHC results in three discordant cases (Validation cohort). There were three discordant cases between MMR‐IHC and MSI‐PCR. (A), (B) Breast cancer (Case # 175, # 200), and (C) lung cancer (Case # 211). The left column shows the shift changes of the five microsatellite markers in the MSI‐PCR for each tumor and its corresponding buffy coat as a control. Right column shows the results of MMR‐IHC images. Original magnification ×100, scale bar 100 μm. In case # 175, there were three shifts (arrows) and four shifts in case # 200 and # 211 by MSI‐PCR. In these three cases, MMR‐IHC revealed no loss of nuclear staining of four proteins (MLH1, MSH2, MSH6, PMS2). All four MMR proteins showed positive nuclear staining in all cases (pMMR)

Paired MSI‐PCR and MMR‐IHC analysis of 319 tumor samples revealed concordant results in 313 of 319 (98.1%), namely MSS/pMMR (281/319: 88.1%) and MSI‐H/dMMR (32/319: 10.0%) (Table 1). Thus, the results of MSI‐PCR and MMR‐IHC were highly concordant, as expected. However, discordant results were observed in six of 319 cases (1.9%) (Table 1). Of these six cases, three (two breast cancers, one lung cancer) were determined as MSI‐H by MSI‐PCR but showed clear nuclear staining of four MMR proteins in any region (Figure 2, Table 2).

**FIGURE 3:**
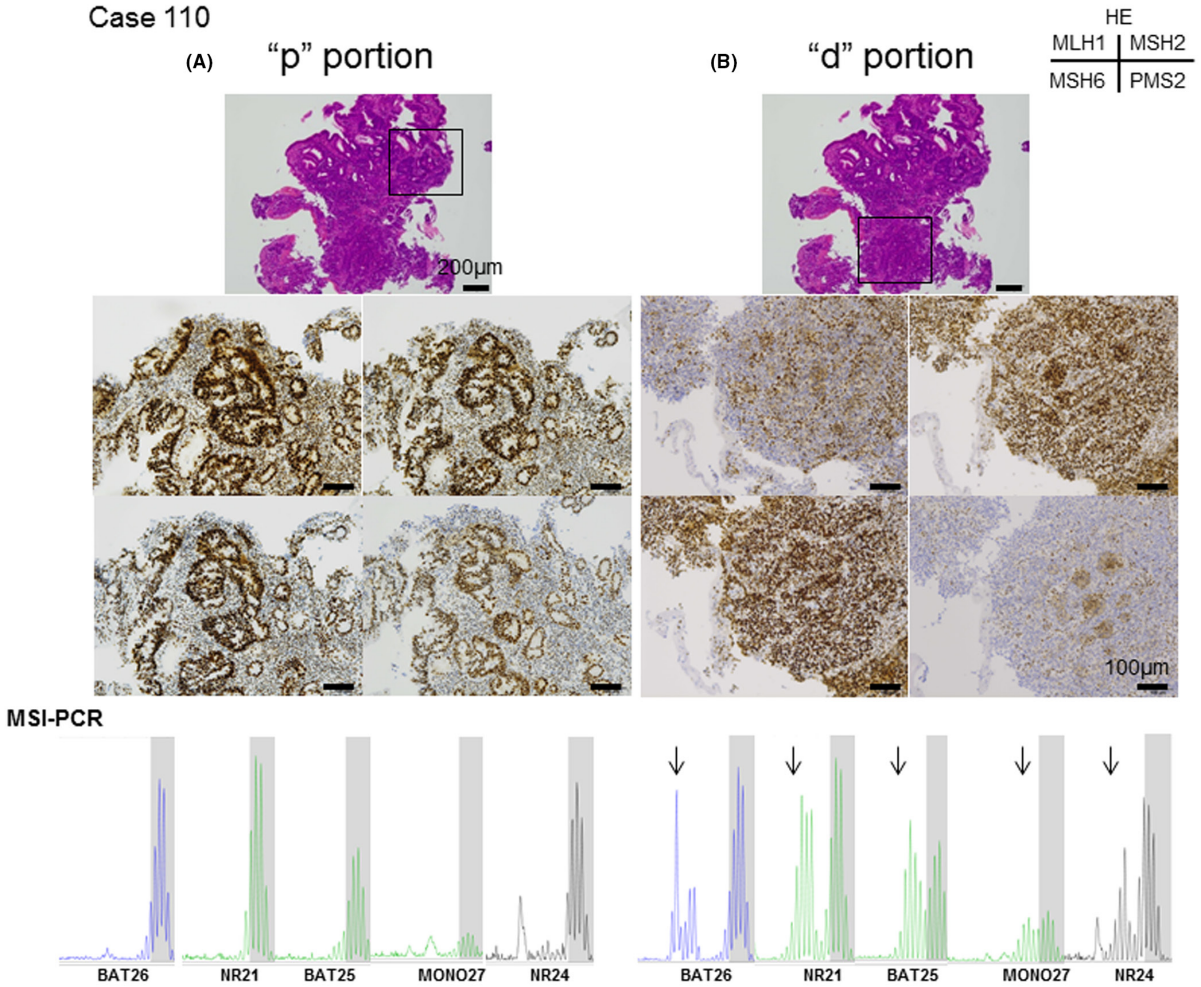
Representative image of MMR‐IHC of mosaic pattern (Case # 110: gastric cancer) and corresponding results of MSI‐PCR for each portion. A mosaic pattern in tumor containing both pMMR and dMMR portions was detected by IHC. (A) In gastric cancer, a pMMR portion by IHC is shown. Original magnification ×20, scale bar 200 μm: HE staining at the top of the figure. Original magnification ×40, scale bar 100 μm: MMR IHC staining. All four MMR‐IHC proteins (MLH1, MSH2, MSH6, PMS2) showed positive nuclear staining. (B) In gastric cancer, dMMR portion by IHC is shown. Original magnification ×20, scale bar 200 μm: HE staining at the top of the figure. Original magnification ×40, scale bar 100 μm: MMR IHC staining. MLH1 and PMS2 were negative for nuclear staining. (C) Arrows show a positive marker in MSI‐PCR. There were five positive shifts in only dMMR portion in case # 110

The remaining three cases (two gastric cancers, one endometrial cancer) were partially “discordant,” and IHC revealed a mosaic staining pattern, namely mixed pMMR and dMMR (p/d MMR) (Figure 3 and Table 2). As shown in Figure 3, in the “p” portion, nuclear staining of four proteins was apparent, whereas in the “d” portion, nuclear staining of two proteins (MLH1 and PMS2) was lost in tumor cells.

### 3.2 Analysis of three discordant cases by MSI‐NGS

We previously reported MSI‐NGS with MSI calling of amplicon‐targeted sequencing of 76 microsatellite loci.^25^ We performed a validation test using MSI‐NGS in the three discordant cases (Table 3).

**TABLE 3:** The results of MMR status of three methods (MSI‐PCR, MMR‐IHC, and MSI‐NGS) in 6 discordant cases (*Validtion* cohort)

Case	Age	Gender	Cancer type	MSI‐PCR			MMR‐IHC		MSI‐NGS		
				Whole	“p” portion	“d” portion	Status	Loss pattern	Whole	“p” portion	“d” portion
175	64	F	Breast	MSI‐H (3)^*^	N.A.	N.A.	pMMR	None	7.6	N.A.	N.A.
200	33	F	Breast	MSI‐H (4)	N.A.	N.A.	pMMR	None	4.9	N.A.	N.A.
211	65	F	Lung	MSI‐H (4)	N.A.	N.A.	pMMR	None	24.6	N.A.	N.A.
110	66	M	Gastric	MSI‐H (5)	MSS (0)	MSI‐H (5)	p/dMMR (5:5)^**^	MLH1, PMS2	129.7	17.3	136.1
169	65	F	Gastric	MSI‐H (3)	MSS (0)	MSI‐H (4)	p/dMMR (3:7)	MLH1, PMS2	18.3	8.5	36.9
306	47	F	Endometrium	MSS (0)	MSS (0)	MSI‐H (2)	p/dMMR (3:7)	MSH6	11.2	7.7	13.3

Abbreviations: MMR‐IHC, mismatch repair‐immunohistochemistry; MSI‐PCR, microsatellite instability‐ polymerase chain reaction; MMR‐NGS, Mismatch repair‐next‐generation sequence; pMMR, proficient MMR; p/d MMR, proficient and deficient MMR; MSI‐H, microsatellite instability high; MSS, microsatellite‐stable; N.A., not applicable.

* Numbers in parentheses indicate the number of positive markers.

** pMMR/dMMR ratio.

The MSI scores of the three discordant cases with a pMMR/MSI‐H status were 7.6 (Case # 175: breast cancer), 4.9 (Case # 200: breast cancer), and 24.6 (Case # 211: lung cancer), and thus all of them were classified as MSS (Table 3). Because these three patients were treated with ICIs on the basis of the MSI‐PCR results, we reviewed their clinical efficiency, but they showed a poor response.

### 3.3 Analysis of three mosaic cases by LCM and MSI‐NGS

In three p/d MMR mosaic cases, LCM was undertaken to enrich the tumor cellularity in each portion and MSI‐PCR and MSI‐NGS were performed (Table 3). In two gastric cancer (Case # 110 and # 169), MSI‐PCR in whole‐tumor tissue revealed MSI‐H. However, after LCM of the pMMR and dMMR portions of tumor tissue according to the guidance of IHC staining, the pMMR portion showed MSS (no peak shift of five markers) and the dMMR portion showed MSI‐H in each case (five in Case # 110 and four in Case # 169) (Table 3). In a gastric tumor (Case # 169), the whole tumor showed MSI in three loci, and the dMMR portion showed MSI in four loci. In endometrial cancer (Case # 306), the whole tumor and pMMR component showed MSS, and only the dMMR portion showed MSI at two loci with a slight shift peak change, which was classified as MSI‐H.

MSI‐NGS in gastric cancer (Case # 110) with a mixed MSI status was classified as MSI‐H for both 129.7 and 136.1 MSI scores for the whole tumor and the dMMR portion, respectively. The pMMR portion was determined as MSS with an MSI score of 17.3.

Endometrial cancer (Case # 306) was MSS, not only in the whole tumor, but also in the pMMR and dMMR portions. The MSI score of the dMMR site was 11.2, which was below the cut‐off value of 40 that defines MSI‐H.

### 3.4 Expanded cohort

MMR‐IHC was performed on 1082 FFPE samples in the Expanded cohort. Loss of at least one MMR protein was observed in 82/1082 (7.6%) (Table 4 and Table S2). Loss of MLH1 and PMS2 was observed in 67/82 (81.7%), while loss of MSH2 and MSH6 was observed in 6/82 (7.3%), loss of PMS2 was observed in 5/82 (6.1%), loss of MSH6 was observed in 3/82 (3.7%), and loss of MLH1, MSH6, and PMS2 was observed in 1/82 (1.2%). In addition, the mosaic pattern was observed in three cases (three endometrial cancers). Loss of MLH1 and PMS2 was observed in dMMR portion of all three mosaic cases (Table 5).

**TABLE 4:** MMR‐IHC in 1082 cases (*Expanded* cohort)

Cancer type	*n*	MMR‐IHC		
		pMMR	dMMR	p/dMMR
Gastric	314	281 (89.5%)	33 (10.5%)	0
Colorectal	275	253 (92%)	22 (8.0%)	0
Breast	12	12 (100%)	0	0
Lung	74	74 (100%)	0	0
Esophagus	13	13 (100%)	0	0
Endometrium	138	110 (79.7%)	25 (18.1%)	3 (2.2%)
Ovary	49	48 (98.0%)	1 (2.0%)	0
Pancreas	15	15 (100%)	0	0
Cervix	2	2 (100%)	0	0
Bladder	10	10 (100%)	0	0
Bile duct	16	15 (94%)	1 (6%)	0
Liver	122	122 (100%)	0	0
Prostate	3	3 (100%)	0	0
Other	39	39 (100%)	0	0
Total	1082	997 (92.1%)	82 (7.6%)	3 (0.3%)

Abbreviations: MMR, Mismatch repair; pMMR, proficient MMR; dMMR, deficient MMR; p/d MMR, proficient and deficient MMR.

**TABLE 5:** MLH1 methylation status and detected variants of four MMR genes (MLH1, MSH2, MSH6, and PMS2) in tumor tissues

Case	Chort	Cancer type	MMR status	Loss pattern	Portion	MLH1 methylation	Somatic variants in MMR genes		
							Gene	Variant	Allelic fraction (%)
175	Validate	Breast	pMMR			N.P.	N.D.	N.D.	N.D.
200	Validate	Breast	pMMR		N.P.	MSH6	p.Met1156Ile	28.6
211	Validate	Lung	pMMR		N.P.	MSH2	p.Gly669Ser	12.8
							MSH2	p.Arg383Ter	7.6
							MSH6	p.Ala339Val	5.3
							MSH6	p.Gly354Glu	5.2
110	Validate	Gastric	d/pMMR	MLH1, PMS2	p	No	N.D.	N.D.	N.D.
					d	Yes	N.D.	N.D.	N.D.
169	Validate	Gastric	d/pMMR	MLH1, PMS2	p	No	N.D.	N.D.	N.D.
					d	No	N.D.	N.D.	N.D.
306	Validate	Endometrium	d/pMMR	MSH6	p	N.P.	N.D.	N.D.	N.D.
					d	N.P.	N.D.	N.D.	N.D.
839	Expanded	Endometrium	d/pMMR	MLH1, PMS2	p	Yes	N.D.	N.D.	N.D.
					d	Yes	N.D.	N.D.	N.D.
905	Expanded	Endometrium	d/pMMR	MLH1, PMS2	p	Yes	N.D.	N.D.	N.D.
					d	Yes	N.D.	N.D.	N.D.
973	Expanded	Endometrium	d/pMMR	MLH1, PMS2	p	Yes	N.D.	N.D.	N.D.
					d	Yes	N.D.	N.D.	N.D.

Abbreviations: MMR, Mismatch repair; pMMR, proficient MMR; dMMR, deficient MMR; p/d MMR, proficient and deficient MMR; N.P., Not performed; N.D., Not detected Variant allelic fraction >5%.

### 3.5 Methylation analysis and somatic variants of MMR genes

We performed methylation analysis and NGS to reveal the cause of mosaic pattern in MMR‐IHC and discordant results between MMR‐IHC and MSI‐PCR. In gastric cancer (Case # 110) with a mixed pMMR and dMMR, only dMMR portion was methylated. Neither portion was methylated in gastric cancer (Case # 169). Three mosaic cases of endometrial cancer (Case # 839, # 905, and # 973) which were loss of MLH1 and PMS2 in MMR‐IHC was methylated both pMMR and dMMR portion. In addition, we performed NGS to examine the somatic variants of four MMR genes (MLH1, MSH2, MSH6, PMS2) in tumor tissues. In lung cancer (Case # 211) detected four somatic variants of MMR genes, and one of which was truncated variant (MSH2 p. Arg383Ter) with low allelic frequency. Low allelic fraction in tumor may affect protein expression.

## 4 DISCUSSION

The introduction of ICIs has resulted in remarkable progress in cancer treatment. In May 2017, pembrolizumab was approved for the treatment of MSI‐H or dMMR unresectable or metastatic solid tumors. Thus, it is critical to correctly assess the status of MMR in tumors.

MSI‐PCR has been the gold standard for testing. In fact, it is mandatory to conduct MSI‐PCR prior to ICI treatment and for reimbursement of government health insurance in Japan. Furthermore, MMR‐IHC is widely performed as a simple method in many laboratories. The concordance rate between the two methods is ~ 90% according to previous reports.^17^, ^20^, ^21^, ^22^, ^23^, ^24^ In these reports, there were a few discordant cases between the two methods, but detailed studies on these cases have not been conducted.

Previous reports indicated that a low tumor rate in endometrial cancer could be the cause of discrepancies between the two analyses.^17^, ^32^ Chapusot et al. reported inconsistencies between the findings of MSI‐PCR and MMR‐IHC in one case among 44 colorectal cancers, demonstrating the intratumoral heterogeneity of MMR‐IHC. Furthermore, macrodissection according to MMR‐IHC staining results and reanalyses have led to concordant results between the two analyses.^33^ In addition, Stelloo et al. also reported that methylation status varies within a tumor, resulting in different MSI statuses.^17^ However, none of these reports used LCM to isolate regions and perform a comprehensive NGS analysis of a large number of microsatellite loci to reassess and compare MSI status.

In this study, we examined the reasons for these discrepancies using LCM and MSI‐NGS, and clarified the usefulness of IHC.

In the Validation cohort, MSI‐PCR and MMR IHC showed high accuracy (313/319: 98.1%), specificity (281/282: 99.6%), and sensitivity (32/37: 86.5%), as expected. However, discrepant results were observed in six cases (6/319: 1.9%). There are two possible reasons for these discrepancies: the ambiguous results of MSI‐PCR assays, and the heterogeneous status of MSI within the tumor.

The first three cases (Cases # 175, # 200 and # 211) were judged as MSI‐H by MSI‐PCR but as pMMR by IHC. Because these are completely contradictory results, another method is required to determine which is accurate. We employed deep sequencing by NGS as the third approach. In our previous report, we examined 76 microsatellite loci instead of five in MSI‐PCR using this MSI‐NGS with MSIcall method.^25^ MSI‐NGS has a lower requirement for computing resources to conduct analyses and a shorter turnaround time, and quantitatively calculates the MSI‐score and evaluates the MSI status without subjectivity. This confirmed MSS in all three cases (Case # 175, # 200, and #211) with scores of 7.6, 4.9, and 24.6 (cut‐off value: 40). When we examined the patterns of these cases by MSI‐PCR, the peak shifts were ambiguous (Figure 2). Therefore, the MSI‐PCR results should be considered as false‐positive. In fact, ICI treatment results were poor in all three cases (data not shown).

Among the other three discordant cases (Case # 110, # 169, and # 306), whole‐tumor MSI‐PCR revealed MSI‐H in two cases (Case # 110, # 169), and MSS in one case (Case # 306). All cases showed a mosaic pattern by MMR‐IHC. Because IHC evaluates the entire tumor, it is easy to determine the diffuseness or localized foci of protein expression. We were able to microdissect individual portions of IHC‐stained and ‐unstained tissue and rechecked our findings by MSI‐PCR. Using this method, the intratumoral heterogeneity of MMR became evident in all three cases (Case # 110, # 169, and # 306). We previously reported that MSI status in multiple gastric cancers is completely different because of the methylation status of MLH‐1 and other proteins, which may explain the intratumoral heterogeneity of MMR.^27^

In the case of endometrial cancer (Case # 306), MSI‐PCR of DNA extracted from the entire tumor showed MSS. However, MMR‐IHC revealed the dMMR portion that was microdissected and was positive for two markers (2/5) of MSI‐PCR. There are reports that the microsatellite marker shift of endometrial cancer is minimal and is less than that in MSI‐H colorectal cancer,^32^, ^34^ thus leading to difficulties in assessing MMR in endometrial cancer. In this case, the opportunity to administer ICIs could have been missed if MSI‐PCR was undertaken in the entire tumor. Therefore, we indicated the obvious utility of IHC in assessing MMR status.

Following sequencing by NGS, in two of three cases (Case # 110 and # 169), MSI‐scores by MSI‐NGS were significantly higher in the dMMR portion than in the pMMR portion and were judged as MSI‐H in only Case # 110 of gastric cancer. In determining MSI status, MSI‐NGS may be useful for ambiguous MSI‐PCR results, but may not work as well for mosaic cases.

For a more detailed study, we performed methylation analysis and NGS to detect somatic variants of MMR genes, including three additional mosaic cases of uterine cancer in the Expanded cohort. Methylation analysis revealed that gastric cancer Case #110 had different MSI status depending on the methylation status. This supports our previous report.^27^ Endometrial cancers (Case # 839, # 905 and # 973) showed methylation at both pMMR and dMMR tumor portion. The methylation rate of tumor cells may be affected by the expression of the MMR protein.

Somatic variants analysis of the MMR gene using NGS, a truncating variant in MSH2 was detected in lung cancer (Case # 211). With a low variant allelic fraction of 7.6% which indicates subclone variant. This reason may reflect discordant result between MSI‐PCR and MMR‐IHC. We detected six mosaic cases (two gastric cancer, four endometrial cancer) in this study, the organ‐specific possibility needs to be verified in a larger number of cases.

Assessing MMR protein expression with IHC is very useful because it is cost‐effective and is widely available in general hospitals, and it also has the ability to determine the affected MMR genes. In fact, we conducted MMR‐IHC in more than 1000 cases as the Expanded cohort. It has maximum benefit in cases that show intratumoral heterogeneity of MMR status, as in this study, and can provide a therapeutic opportunity for ICIs that may have otherwise been missed.

MMR‐IHC is also effective in picking up patients with suspected Lynch syndrome. In 1401 samples performed IHC, 114 samples of 101 patients with dMMR were reviewed the medical history of Lynch‐related tumors in the electronic medical record, 22% (22/101) of them had synchronous and/or metachronous Lynch syndrome‐associated tumors (Sup table 3).

Recently, regarding the use of ICIs with IHC, the Ventana MMR RxDx panel was approved by the Food and Drug Administration in April 2021 as a companion diagnostic to the anti‐PD‐1 antibody dostarlimab^35^ in adult patients with recurrent or advanced dMMR/MSI‐H endometrial cancer that has progressed on or after treatment with a platinum‐containing regimen. MMR‐IHC has not yet been approved for a diagnostic testing kit that has the potential to be used in all solid tumors to determine the use of pembrolizumab in Japan, and early expansion of this indication is desired.

## CONFLICT OF INTEREST DISCLOSURES

The authors have no disclosures.

## ETHICAL STATEMENT

This study was approved by the institutional review board at our hospital (G2018–1, G2019–5). Written informed consent was obtained from all patients who participated in this study.

## AUTHOR CONTRIBUTION

Kenji Amemiya carried out conceptualization, data curation, investigation, funding acquisition, methodology, and writing—original draft of the manuscript. Yosuke Hirotsu was involved in investigation, data curation, funding acquisition, methodology, and writing—reviewing and editing of the manuscript. Yuki Nagakubo, Shunsuke Watanabe, and Saki Amemiya carried out investigation. Hitoshi Mochizuki carried out methodology. Toshio Oyama was involved in resources, methodology, and investigation, Tetsuo Kondo contributed to writing‐review, editing, and supervision. Masao Omata carried out conceptualization, funding acquisition, project administration, and supervision. All authors have read and agreed to the final version of this manuscript.

## Supporting information


Table S1

Table S2

Table S3

## Data Availability

Data sharing is not applicable to this article as no new data were created or analyzed in this study.
